# NUTRITIONAL RISK PREDICTS HEALTH SERVICES UTILIZATION AND DEATH OVER 1 YEAR: RESULTS FROM THE UAB STUDY OF AGING II

**DOI:** 10.1093/geroni/igz038.1257

**Published:** 2019-11-08

**Authors:** David R Buys, Richard E Kennedy, Yue Zhang, Julie Locher, Cynthia J Brown

**Affiliations:** 1 Mississippi State University, Starkville, Mississippi, United States; 2 University of Alabama at Birmingham, Birmingham, Alabama, United States

## Abstract

Nutritional risk has been demonstrated to be associated with poor health outcomes, increased risk of health services utilization (HSU), and mortality among older adults. The aim of this study was to assess the prospective relationship between nutritional risk; HSU focusing separately on emergency department visits, hospitalization, and nursing home admission; and mortality. Using the University of Alabama-Birmingham Study of Aging II, we examined this relationship among 419 community-dwelling older Alabamians (75+years). We used the Mini-Nutrition Assessment (MNA), a well-validated nutritional risk assessment, which classifies individuals as either well-nourished, at-risk or malnourished, collected at baseline. We assessed HSU by asking about healthcare encounters since the last monthly follow-up call for 12 months and verified death with family reports and official documents. We completed univariate, bivariate, and Cox proportional hazards regression analyses with one-year of follow-up data, adjusting for social support, social isolation, comorbidities, and demographic variables. Accounting for covariates, being either at-risk or malnourished, relative to well-nourished, was associated with emergency department visits (HR: 1.30, 95% CI:1.14,1.48), hospitalization (HR: 1.58, 95% CI:1.37,1.82), nursing home admission (HR: 8.94, 95% CI:3.99,20.02), and mortality (HR: 1.90, 95% CI:1.25,2.88). These findings underscore the growing awareness that nutritional risk, particularly for older adults, is a significant factor affecting their well-being and particularly their ability to continue living in the community. Nutrition assessment, interventions, and services for community-dwelling older adults may lead to a reduction in health care utilization, particularly nursing home placement, and ultimately to reduced healthcare costs to families and taxpayers.

